# Technical report on the first clinical use of a bioabsorbable PGA spacer in HDR brachytherapy for recurrent cervical cancer

**DOI:** 10.1093/jrr/rraf053

**Published:** 2025-08-22

**Authors:** Kae Okuma, Tomomichi Kiyomatsu, Hirotoshi Takiyama, Akane Yoshiba, Koji Inaba, Tairo Kashihara, Kana Takahashi, Madoka Sakuramachi, Ayaka Nagao, Hiroshi Igaki

**Affiliations:** Department of Radiation Oncology, National Cancer Center Hospital, 5-1-1 Tsukiji, Chuo-ku, Tokyo 104-0045, Japan; Department of Surgery, National Center for Global Health and Medicine, 1-21-1 Toyama, Shinjuku-ku, Tokyo 162-8655, Japan; Radiation Oncology, QST Hospital, National Institutes for Quantum and Radiological Science and Technology, 4-9-1 Anagawa, Inage-ku, Chiba-shi, Chiba 263-8655, Japan; Department of Radiation Oncology, National Cancer Center Hospital, 5-1-1 Tsukiji, Chuo-ku, Tokyo 104-0045, Japan; Department of Radiation Oncology, National Cancer Center Hospital, 5-1-1 Tsukiji, Chuo-ku, Tokyo 104-0045, Japan; Department of Radiation Oncology, National Cancer Center Hospital, 5-1-1 Tsukiji, Chuo-ku, Tokyo 104-0045, Japan; Department of Radiation Oncology, National Cancer Center Hospital, 5-1-1 Tsukiji, Chuo-ku, Tokyo 104-0045, Japan; Department of Radiation Oncology, National Cancer Center Hospital, 5-1-1 Tsukiji, Chuo-ku, Tokyo 104-0045, Japan; Department of Radiation Oncology, National Cancer Center Hospital, 5-1-1 Tsukiji, Chuo-ku, Tokyo 104-0045, Japan; Department of Radiation Oncology, National Cancer Center Hospital, 5-1-1 Tsukiji, Chuo-ku, Tokyo 104-0045, Japan

**Keywords:** cervical cancer, reirradiation, high-dose-rate brachytherapy, PGA spacer, biodegradable spacer, small bowel dose reduction

## Abstract

In recurrent gynecologic malignancies following prior pelvic irradiation, definitive radiation therapy is often precluded by cumulative dose constraints to adjacent organs at risk (OARs), and patients may be left with only highly invasive surgical options such as total pelvic exenteration. While some institutions have explored displacement techniques such as artificial ascites or hyaluronic acid gel injection, these approaches are not widely adopted and frequently fail to ensure consistent and stable separation of OARs. We report the first clinical use of Neskeep®, a bioabsorbable polyglycolic acid (PGA) spacer, in high-dose-rate (HDR) brachytherapy for recurrent cervical cancer after prior pelvic radiation. A woman in her 40s with prior hysterectomy and HDR brachytherapy for cervical intraepithelial neoplasia grade III developed vaginal stump recurrence 4 years later. Laparoscopic placement of the PGA spacer was performed to achieve durable displacement of the small bowel, followed by eight fractions of HDR brachytherapy. Hyaluronic acid gel was also injected during each fraction to displace the bladder and rectum. The spacer maintained position and volume throughout treatment without complications. Dose–volume analysis showed a marked reduction in small bowel *D*_₂cc_ (mean equivalent dose in 2 Gy fractions (EQD₂): 121.6 cGy) compared to the initial treatment (606.0 cGy), while the spacer itself received a mean *D*_₂cc_ of 690.3 cGy. MRI confirmed complete response at 2 months, with no adverse events observed at that time point. The PGA spacer enabled safe, curative reirradiation in a case that would otherwise be unsuitable for further radiation therapy.

## INTRODUCTION

Brachytherapy plays a crucial role in achieving local control in gynecologic malignancies. In cervical cancer in particular, the combination of external beam radiation therapy (EBRT) and brachytherapy constitutes the standard of care [1, 2]. Brachytherapy allows for the delivery of high radiation doses to the tumor while sharply reducing exposure to surrounding normal tissues, making it effective even in the setting of recurrence [3–6]. However, achieving adequate sparing of adjacent organs at risk (OARs), such as the small bowel, remains a critical challenge—especially in reirradiation settings.

The PGA spacer (Neskeep®, Alfresa Pharma Co., Osaka, Japan) is an innovative bioabsorbable medical device developed in Japan to physically separate tumors from adjacent OARs. Composed of a nonwoven fabric made of highly water-absorbent polyglycolic acid (PGA), the spacer maintains its thickness and structural integrity for ~3 months before undergoing hydrolytic degradation and absorption. Its safety and effectiveness have been demonstrated primarily in particle therapy, including proton and carbon-ion radiation [7–11]. In particular, the PGA spacer has shown utility in cases involving close proximity to the gastrointestinal tract, enabling dose escalation while minimizing the risk of severe toxicity.

The PGA spacer was approved for insurance reimbursement in Japan for use in particle therapy in December 2019, and more recently, in September 2023, for general use in radiotherapy. However, clinical experience with the PGA spacer outside of particle therapy remains limited, and to our knowledge no previous reports have described its application in high-dose-rate (HDR) brachytherapy. Herein, we report the first clinical application of the PGA spacer in HDR brachytherapy for recurrent cervical cancer following prior radiotherapy. In this case, laparoscopic placement of the PGA spacer between the recurrent tumor and the adjacent small bowel enabled safe dose delivery while avoiding excessive exposure to OARs. This technical report highlights the feasibility and clinical potential of using the bioabsorbable PGA spacer in gynecologic reirradiation settings.

## MATERIALS AND METHODS

The patient was a woman in her 40s who initially underwent cervical conization for cervical intraepithelial neoplasia grade III (CIN III). Despite treatment, high-grade squamous intraepithelial lesions (HSILs) persisted on cytology, and she subsequently underwent robot-assisted total hysterectomy. Final pathology revealed CIN II at the vaginal resection margin between the 3 and 5 o’clock positions.

**Fig. 1:**
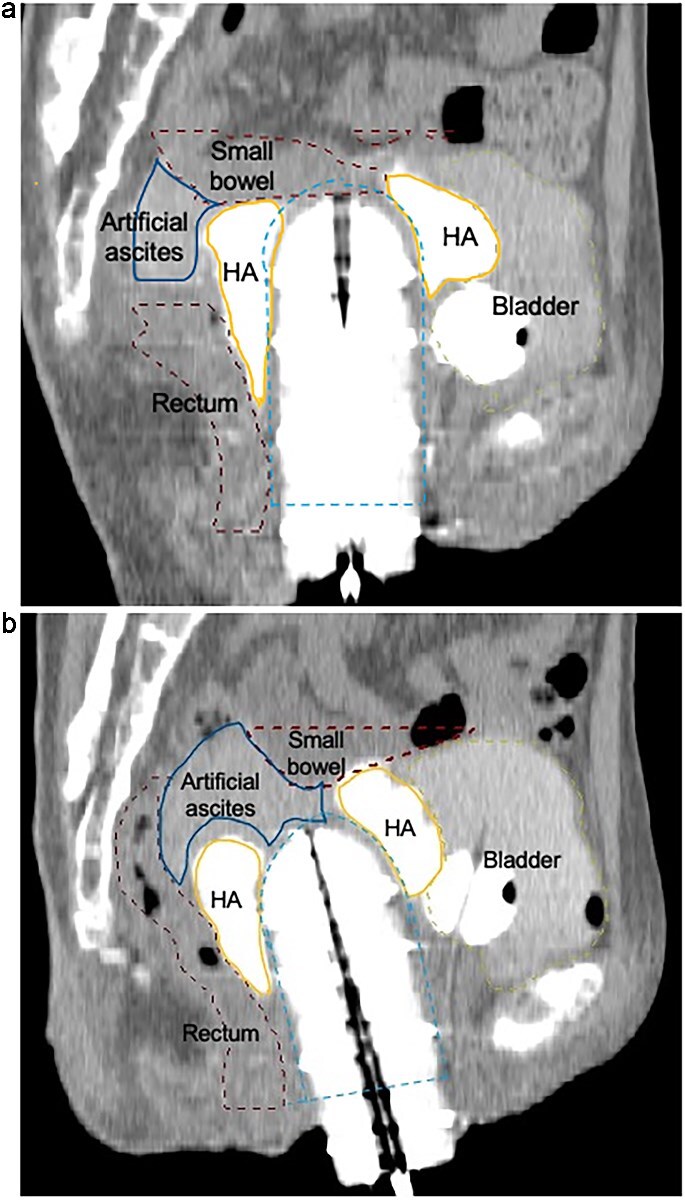
(a) Sagittal CT image acquired during the initial HDR brachytherapy session. Artificial ascites (blue contour, lateral) was instilled intraperitoneally to separate the small bowel (red dashed contour, superior) from the high-dose region, and hyaluronic acid (HA) gel (orange contour, bilateral anterior) was injected into the vesicovaginal and rectovaginal spaces to displace the bladder and rectum. Despite these efforts, the small bowel remained in close proximity to the target volume. (b) Sagittal CT image acquired during the fourth fraction of the initial HDR brachytherapy. To reduce radiation exposure to adjacent organs, HA gel (orange contour, bilateral anterior) was injected into both the vesicovaginal and rectovaginal spaces, and artificial ascites (blue contour, lateral) was instilled to displace the small bowel (red dashed contour, superior). In this fraction, a greater cranial shift of the small bowel was observed compared to earlier fractions, indicating improved displacement efficacy. The bladder and rectum are delineated by yellow (anterior) and dark red (posterior) dashed contours, respectively. Line styles and anatomical positions are provided alongside color for accessibility.

**Fig. 2:**
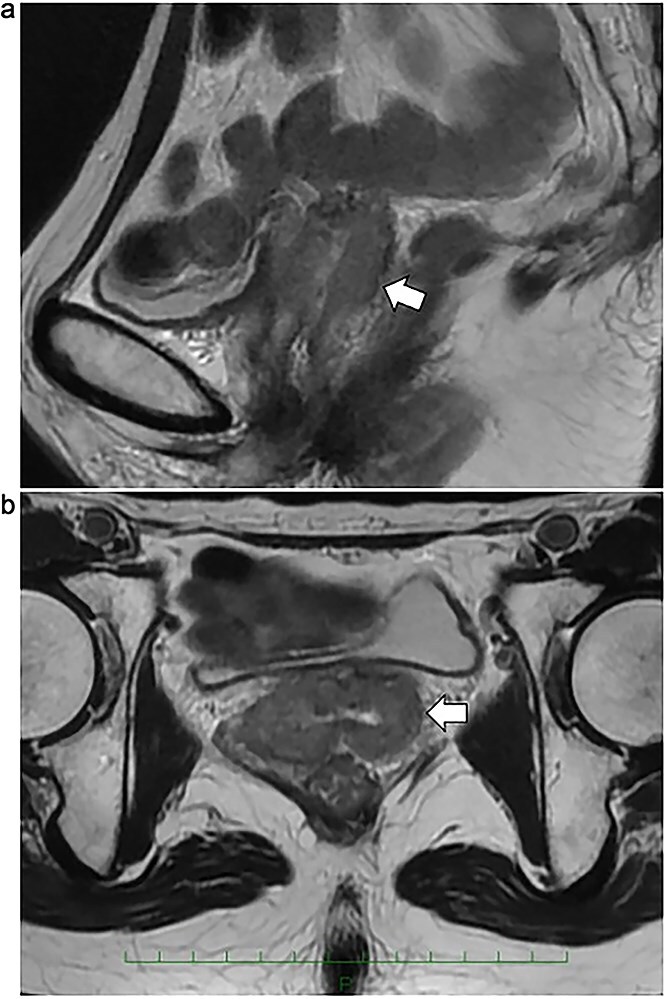
(a) Sagittal *T*_2_-weighted MRI prior to reirradiation. A recurrent tumor (arrow) is visualized along the vaginal cuff, exhibiting contact with the adjacent small bowel. Adhesion between the tumor and bowel was suspected based on imaging. (b) Axial *T*_2_-weighted MRI demonstrating circumferential thickening of the recurrent lesion (arrow) along the entire vaginal wall. No evidence of pelvic lymph node or distant metastasis was identified on MRI, CT, or PET-CT.

Three months postoperatively, HSIL recurred at the vaginal cuff, and colposcopy revealed a 3-cm iodine-unstained lesion. Five months after surgery, HDR brachytherapy was administered (6 Gy × 5 fractions) using a vaginal cylinder applicator, with the clinical target volume (CTV) defined as the vaginal submucosa within 5 mm depth. To reduce radiation exposure to the bladder and rectum, hyaluronic acid (HA) gel was injected into the vesicovaginal and rectovaginal spaces during each fraction. These anatomical compartments are relatively enclosed, and ~20 cc of HA gel was sufficient to achieve effective separation of adjacent organs. To further reduce radiation exposure to the small bowel, normal saline was infused into the peritoneal cavity as artificial ascites. Since the peritoneal cavity is a larger and less confined space, the same volume of HA gel would have dispersed without achieving meaningful bowel displacement. In contrast, the use of a larger volume of normal saline allowed for more effective cranial displacement of mobile small bowel from the high-dose region directly above the vaginal cuff (Fig. 1). Although a complete response was achieved, CIN III persisted, and 4 years later, local recurrence of squamous cell carcinoma at the vaginal cuff was histologically confirmed. Imaging studies (CT, PET-CT and MRI) showed no evidence of lymph node or distant metastasis, and the recurrence was deemed isolated and local. Surgical resection was considered; however, given the patient’s prior surgical and radiation history, there was a high likelihood that total pelvic exenteration would be required due to anticipated adhesions and the potential risk of further recurrence. Given the prior history of EBRT and HDR brachytherapy, further EBRT posed a risk of exceeding the dose constraints for the bladder, rectum and small bowel. MRI demonstrated a circumferential recurrent tumor extending along the entire vaginal vault, with a thickness > 1 cm and suspected contact or adhesion to the small bowel (Fig. 2). Therefore, HDR brachytherapy alone was selected for reirradiation.

As in the initial treatment, HA gel was injected into the vesicovaginal and rectovaginal spaces during each fraction to reduce the dose to the bladder and rectum. The gel preparation used was a commercially available intra-articular HA formulation, repurposed for this use under institutional approval. However, the tumor–small bowel interface was indistinct on imaging, and stable placement of HA gel within the peritoneal cavity was considered unfeasible. Moreover, artificial ascites used in the initial treatment could not always be retained in a specific region of the peritoneal cavity, limiting its effectiveness. In addition, due to rapid absorption, there was a risk that the infused saline would be reabsorbed before the start of treatment. Therefore, laparoscopic placement of a bioabsorbable PGA spacer was planned. After consultation with a radiation oncologist experienced in the use of the PGA spacer during particle therapy, a gastrointestinal surgeon skilled in laparoscopic spacer placement was identified, and the procedure was scheduled to be performed at a collaborating institution 2 weeks later. The patient was preoperatively counseled that spacer placement could be aborted in the presence of dense adhesions, due to the potential risk of residual tumor, peritoneal dissemination or infection. Diagnostic laparoscopy was initially planned to assess the presence of adhesions between the recurrent tumor and adjacent small bowel. If dense adhesions had been encountered, the procedure would have been terminated without spacer placement. However, since no such adhesions were present, laparoscopic placement was deemed feasible and performed. Laparoscopy was selected over open surgery to minimize invasiveness, considering the potential need to abandon the procedure. Open surgery is generally considered only in cases requiring extensive adhesiolysis or where laparoscopic access is anatomically restricted, although this approach was not employed in the present case.

## RESULTS

The laparoscopic procedure was performed under general anesthesia. Intraoperatively, no tumor exposure into the peritoneal cavity or adhesions with the small bowel were observed, and no peritoneal dissemination was detected. A 15-mm-thick sheet of the PGA spacer was trimmed to 10 × 10 cm, inserted through a Pfannenstiel incision and sutured in place. The total operative time was 1 hour and 50 minutes. Postoperatively, the patient experienced a low-grade fever for 3 days and transient groin pain caused by contact between the spacer edge and the abdominal wall, which was managed with analgesics.

HDR brachytherapy was initiated 7 days after surgery. A total of eight fractions (6 Gy per fraction) were administered over 6 weeks on an outpatient basis, at a frequency of one to two sessions per week. The CTV encompassed the entire recurrent lesion along the vaginal wall. Treatment was delivered using a vaginal cylinder applicator in combination with several 5 Fr plastic interstitial needles. All the procedures were performed under sacral nerve block and intravenous sedation, and were well tolerated.

Hyaluronic acid (HA) gel spacers were injected into the vesicovaginal and rectovaginal spaces at each fraction to reduce radiation exposure to the bladder and rectum. The gel was composed of three syringes of purified sodium hyaluronate (hyaluronic acid Na injection 25 mg ‘Meiji’, Meiji Seika Pharma), mixed with saline and contrast agent.

**Fig. 3:**
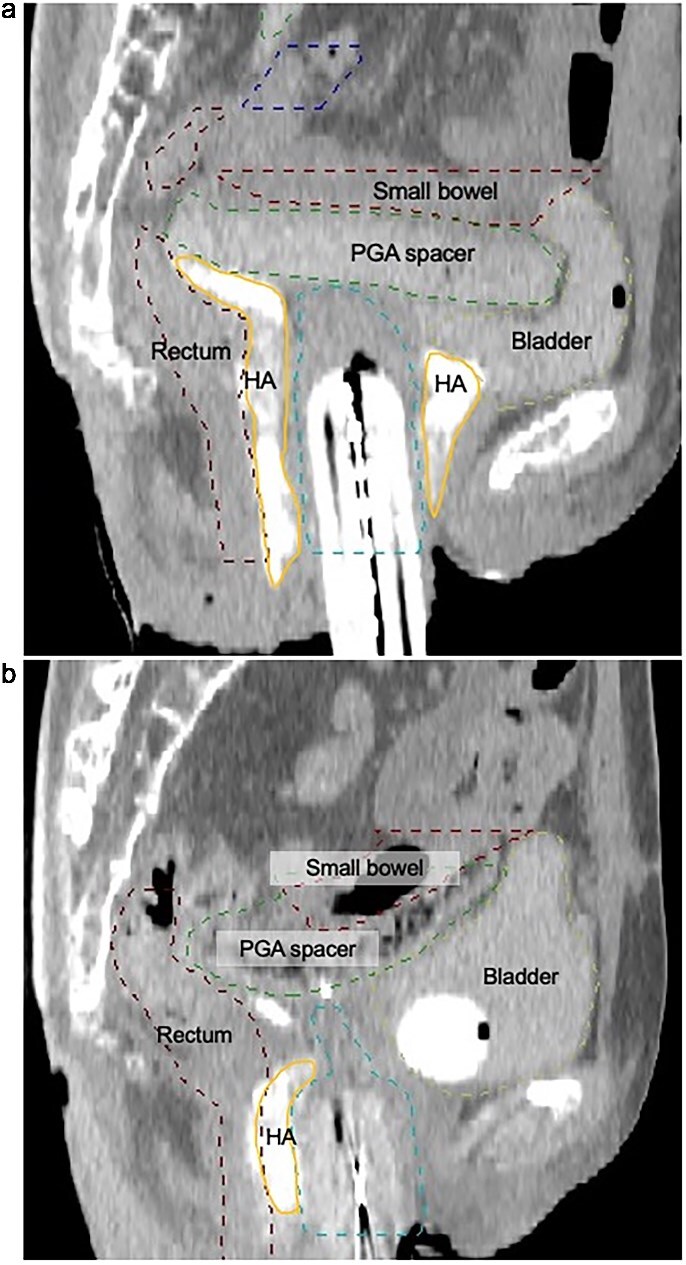
(a) Sagittal CT image acquired during the first fraction of the reirradiation HDR brachytherapy. A bioabsorbable PGA spacer (PGA spacer; green dashed contour, central) was laparoscopically placed between the recurrent tumor and the small bowel (red dashed contour, superior). Hyaluronic acid (HA) gel (HA; orange contour, bilateral anterior) was injected between the bladder and rectum to reduce dose exposure to these organs. The PGA spacer successfully created separation from the small bowel, enabling safe delivery of curative doses. The bladder and rectum are outlined with yellow (anterior) and dark red (posterior) dashed contours, respectively. (b) Sagittal CT image from the eighth fraction of HDR brachytherapy during reirradiation. The bioabsorbable spacer (PGA spacer; green dashed contour) shows partial degradation in its central region, yet maintains sufficient separation between the recurrent tumor and the small bowel (red dashed contour, superior). The spacer continues to fulfill its intended role of protecting adjacent organs. HA gel (HA; orange contour) was injected between the rectum and the applicator to reduce the rectal dose. The rectum and bladder are delineated by dark red (posterior) and yellow (anterior) dashed contours, respectively. Line styles and anatomical positions are provided alongside color for accessibility.

CT imaging demonstrated that the PGA spacer remained stable throughout the treatment course, though gradual central resorption was observed (Fig. 3). The spacer provided consistent cranial displacement of the small bowel across all eight fractions, facilitating safe delivery of curative doses. Figure 1 illustrates sagittal CT images from the initial brachytherapy course using artificial ascites. Compared to the PGA spacer, artificial ascites showed inconsistent bowel displacement between fractions, underscoring the limitations of this method.

**Fig. 4:**
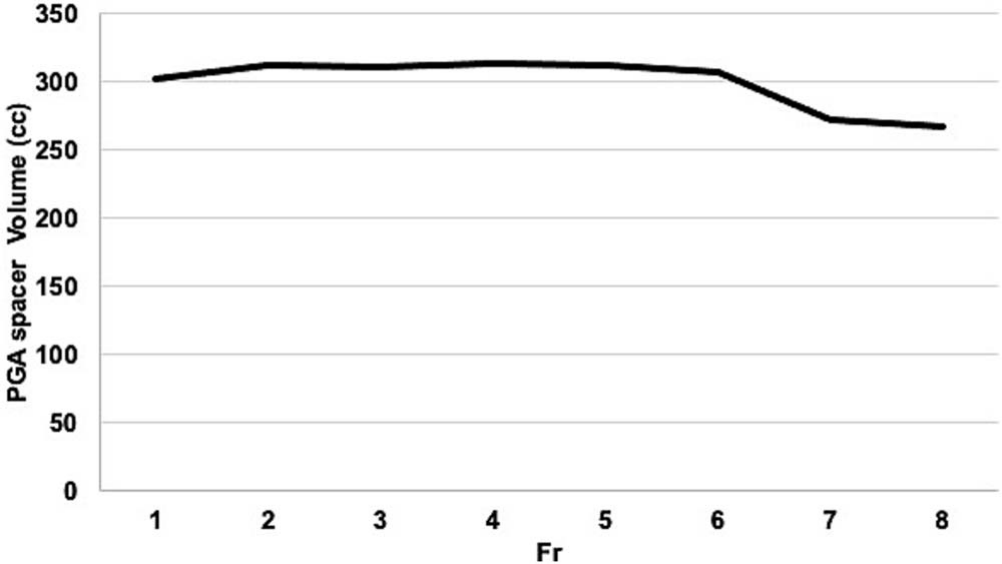
Temporal profile of the PGA spacer volume across eight HDR brachytherapy fractions. The volume of the bioabsorbable PGA spacer was delineated on pretreatment CT images obtained for each fraction and calculated using the treatment planning system. The spacer preserved a volume > 300 cc through the sixth fraction, with a modest decline to 271.9 and 266.9 cc during the seventh and eighth fractions, respectively. These findings indicate satisfactory volumetric stability and geometric integrity of the spacer throughout the treatment course.

**Fig. 5:**
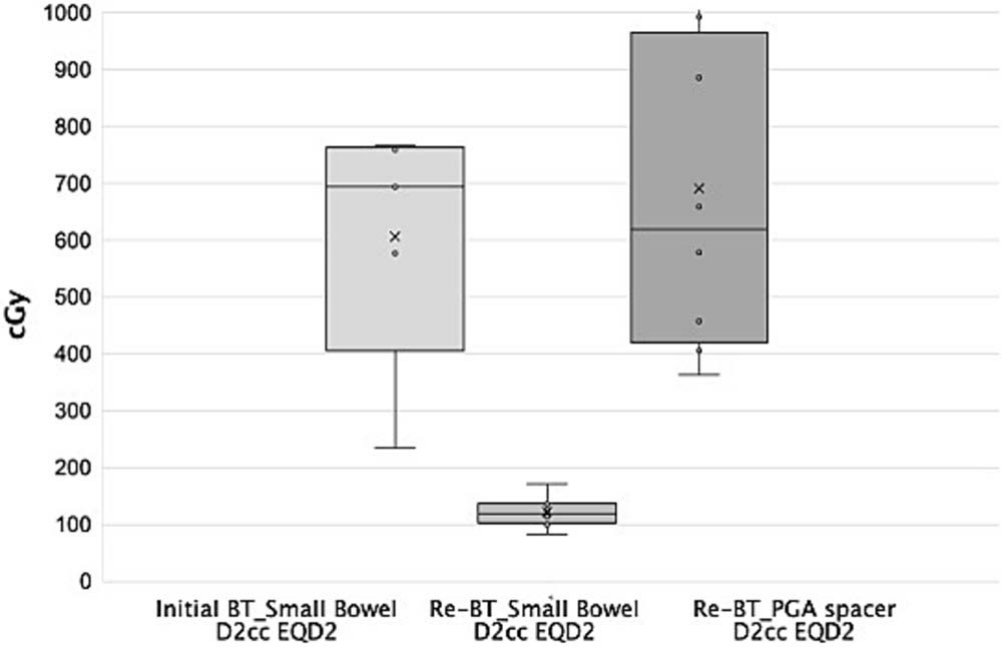
Box plots comparing *D*_2cc_ EQD2 doses for the small bowel and the PGA spacer between the initial and reirradiation HDR brachytherapy sessions. ‘Initial BT_Small Bowel D2cc EQD2’ represents the small bowel dose during the first course of brachytherapy; ‘Re-BT_Small Bowel D2cc EQD2’ indicates the small bowel dose during reirradiation with the PGA spacer placement; and ‘Re-BT_PGA spacer D2cc EQD2’ denotes the maximum dose delivered to 2 cc of the PGA spacer. The use of the PGA spacer enabled substantial reduction in small bowel dose while permitting high-dose delivery to the target.

The spacer volume remained relatively stable, decreasing slightly from 307.2 cc at the first fraction to 266.9 cc at the eighth, confirming adequate shape retention throughout treatment (Fig. 4). Dose–volume histogram analysis showed a significant reduction in small bowel *D*_₂cc_ during reirradiation (mean equivalent dose in 2 Gy fractions (EQD₂): 121.8 cGy; range: 83.0–170.5 cGy) compared to the initial HDR brachytherapy (mean EQD₂: 606.0 cGy; range: 235.0–766.0 cGy). Meanwhile, the PGA spacer itself received a mean *D*_₂cc_ of 690.3 cGy (range: 362.0–1184.0 cGy), suggesting that it effectively absorbed a substantial proportion of the dose otherwise delivered to the bowel (Table 1 and Fig. 5). To statistically validate the observed reduction in small bowel *D*_₂cc_ during reirradiation, a Wilcoxon signed-rank test was performed comparing the values without and with the PGA spacer. The analysis demonstrated a significant decrease in *D*_₂cc_ (median: 694.0 cGy vs 114.0 cGy; *P* = 0.043), confirming the dosimetric benefit of spacer placement.

**Table 1:** Summary of equivalent dose in 2 Gy fractions (EQD2, Gy) for the minimum dose to the most exposed 2 cc (*D*_2cc_) of the small bowel and the bioabsorbable PGA spacer during initial and reirradiation high-dose-rate brachytherapy^a^

	Average (cGy)	Median (cGy)	Min (cGy)	Max (cGy)	SD (cGy)	Range (cGy)
Initial BT: small bowel *D*_2cc_	606.0	694.0	235.0	766.0	±221.0	531.0
Re-BT: small bowel *D*_2cc_	121.6	119.0	83.0	170.5	±26.9	87.5
Re-BT: the PGA spacer *D*_2cc_	690.3	618.0	362.0	1184.0	±299.8	822.0

^a^Compared with the initial course, a marked reduction in small bowel *D*_2cc_ was achieved during reirradiation, whereas the PGA spacer received a substantial portion of the therapeutic dose, indicating its effectiveness in dose shielding. BT = brachytherapy.

No acute complications related to spacer placement or brachytherapy were observed, aside from the aforementioned transient groin pain. At 2 months posttreatment, pelvic MRI confirmed complete remission, with no evidence of residual tumor.

These results demonstrate the feasibility and dosimetric benefits of using a bioabsorbable PGA spacer in HDR brachytherapy for recurrent gynecologic malignancy. In particular, the substantial reduction in small bowel dose, combined with the stable positioning and volume retention of the spacer, supports its role as a reliable tool for improving the safety of reirradiation. We next discuss the clinical implications and limitations of this approach.

## DISCUSSION

Reirradiation for recurrent gynecologic malignancies is inherently challenging due to the cumulative dose constraints of surrounding OARs, particularly the small bowel. In the present case, the use of a bioabsorbable PGA spacer enabled a substantial reduction in the small bowel dose (mean *D*_₂cc_: 121.8 vs 606.0 cGy in the initial treatment) and maintained consistent anatomical separation throughout all eight fractions, with no significant complications.

Conventional displacement techniques such as artificial ascites and HA gel injection have been reported to physically separate OARs from the target volume during brachytherapy [7, 8]. However, their clinical adoption remains limited both in Japan and internationally, and their effects are often transient or anatomically insufficient—especially in reirradiation cases with suspected adhesions or minimal peritoneal space. In our institutional practice, HA gel is routinely used to displace the bladder and rectum by injecting it into the vesicovaginal and rectovaginal spaces, which are relatively enclosed and anatomically confined. In contrast, the small bowel resides within the peritoneal cavity, where injected HA gel tends to disperse diffusely without achieving effective displacement.

In the present case, the reason for not using a PGA spacer during the initial course of HDR brachytherapy was not solely the lack of insurance coverage at that time. More importantly, the initial treatment was the patient’s first pelvic irradiation and thus was not affected by cumulative dose constraints. Consequently, we selected artificial ascites, a less invasive and more feasible approach. In contrast, reirradiation required a more stable and reliable method of bowel displacement due to prior radiation exposure and suspected proximity of the small bowel to the target. While the current case involved recurrent disease, we believe that surgically placed PGA spacers may also be beneficial in primary settings where high-dose irradiation is necessary and the target volume lies adjacent to the bowel.

Moreover, laparoscopic placement of the PGA spacer provided stable, reproducible and sustained displacement of the small bowel throughout the entire treatment course. HA gel spacers are routinely injected into more confined anatomical spaces, such as the vesicovaginal and rectovaginal compartments. Given the technical simplicity and reproducibility of this method under transrectal ultrasound guidance, we do not consider the use of PGA spacers in these compartments, and thus a direct comparison with HA gel was not conducted in this study. Additionally, the utility of HA gel for dose reduction in gynecologic brachytherapy has been previously reported from our institution [9].

While Fig. 4 was included to confirm the shape retention of the PGA spacer, it does not directly represent the secured distance from OARs. The instability of artificial ascites is illustrated in Fig. 1a and b, but due to its fluid nature, a precise quantitative assessment was not feasible. Therefore, we believe a broader quantitative comparison of all spacer types is outside the scope of this technical report.

Importantly, surgical resection such as total pelvic exenteration was initially considered as a curative option. However, the patient and clinical team opted for a less invasive approach due to the expected postoperative morbidity and quality-of-life implications. The ability to achieve complete tumor response with spacer-assisted reirradiation highlights the clinical value of this technique in anatomically complex or surgically unfavorable cases.

However, a notable limitation is that PGA spacer placement requires surgical intervention under general anesthesia, even if performed laparoscopically. This requirement limits its feasibility for routine use, especially in initial definitive radiotherapy. In contrast, HA gel and artificial ascites offer the advantage of being minimally invasive and can be administered safely without surgery. These nonsurgical options remain the preferred approach in most primary cases, whereas PGA spacers may be best reserved for anatomically complex reirradiation scenarios where more stable and durable displacement is required.

To our knowledge, this is the first clinical report of the PGA spacer being applied in HDR brachytherapy. The spacer demonstrated favorable shape retention and absorbed a substantial proportion of the delivered dose. These findings support its feasibility and safety, and suggest that the PGA spacer may serve as a valuable tool in expanding safe reirradiation options for recurrent gynecologi malignancies.

## CONCLUSION

The use of the PGA spacer may represent a feasible and effective approach to achieve small bowel dose reduction during HDR brachytherapy for recurrent pelvic tumors, particularly in previously irradiated patients. Further case accumulation and prospective studies will be necessary to validate its clinical utility and define its role in standard practice.

## PRESENTATION AT A CONFERENCE

This work is scheduled to be presented at the 9th Annual Meeting of the Japanese Space-Making Radiation Therapy Group, June 2025, Japan.

## DECLARATION OF GENERATIVE AI AND AI-ASSISTED TECHNOLOGIES IN THE WRITING PROCESS

During the preparation of this work, the authors used ChatGPT (OpenAI) to improve the language, grammar and clarity of the manuscript. After using this tool, the authors reviewed and edited the content to ensure accuracy and appropriateness.

## Data Availability

No datasets were generated or analyzed during the current study.
